# Analysis of the Modified Rankin Scale in Randomised Controlled Trials of Acute Ischaemic Stroke: A Systematic Review

**DOI:** 10.1155/2016/9482876

**Published:** 2016-03-20

**Authors:** Aimie Nunn, Philip M. Bath, Laura J. Gray

**Affiliations:** ^1^Statistics Department, Quanticate, Hitchin SG5 1LH, UK; ^2^Stroke Trials Unit, Division of Clinical Neuroscience, University of Nottingham, Nottingham NG5 1PB, UK; ^3^Department of Health Sciences, University of Leicester, Leicester LE1 7RH, UK

## Abstract

*Background*. Historically, most acute stroke clinical trials were neutral statistically, with trials typically dichotomising ordinal scales, such as the modified Rankin Scale. Studies published before 2007 have shown that preserving the ordinal nature of these scales increased statistical power. A systematic review of trials published since 2007 was conducted to reevaluate statistical methods used and to assess whether practice has changed.* Methods*. A search of electronic databases identified RCTs published between January 2007 and July 2014 in acute ischaemic stroke using an ordinal dependency scale as the primary outcome.* Findings*. Forty-two RCTs were identified. The majority used a dichotomous analysis (25, 59.5%), eight (21.4%) retained the ordinal scale, and nine (19.0%) used another type of analysis.* Conclusions*. Trials published since 2007 still favoured dichotomous analyses over ordinal. Stroke trials, where appropriate, should consider retaining the ordinal nature of dependency scales.

## 1. Introduction

The modified Rankin Scale (mRS) is a 7-level ordered categorical scale capturing levels of patient functional independence following a stroke, with scores ranging from 0 (fully independent) to 6 (dead) [1]. The mRS has been reported to be a valid and reliable endpoint in randomised clinical trials [2] and as such it is a common and recommended outcome measure in acute ischaemic stroke studies [3].

Historically, clinical trials in acute ischaemic stroke have largely been unable to show statistical benefit of therapy over control [4]. This failure has been attributed to multiple causes, including the relevance of laboratory findings to clinical stroke [5], inadequate sample size [6], choice of primary outcome, and its statistical analysis. The majority of trials have previously favoured dichotomous analysis of outcome measures that employ an ordinal scale [7]. However, previous reviews of stroke outcomes have suggested that the choice of analytical methods has been less than optimal [8]. The OAST collaboration published a reanalysis of stroke outcomes using alternative statistical methods in 2007 and showed that methods preserving the ordinal nature of the original data were the most optimal [7]. Ordinal logistic regression (OLR) was shown to provide the most statistically efficient analysis of ordinal outcome scales when the proportional odds assumption was met, permitting trial sample size to be reduced compared to dichotomous analysis [7, 9]. This along with other related works led the European Stroke Organisation Outcomes Working Group to recommend that trialists move away from dichotomous outcomes and chose an analysis approach based on the type of patients to be recruited and the likely mechanism of the intervention to be tested [10].

The primary objective of this systematic review is to provide an updated evaluation of statistical methods used in the analysis of the mRS in clinical trials of acute ischaemic stroke published from 2007 to 2014. Given the recommendations made by the OAST collaboration in 2007, it is pertinent to assess whether these findings have influenced more recent trends in analysis of ordinal outcomes in acute stroke studies.

## 2. Materials and Methods

### 2.1. Search Strategy

Overlapping search strategies were conducted in order to identify a complete list of trials for systematic review. National Centre for Biotechnology Information (NCBI) PubMed, Ovid MEDLINE, and Cochrane Collaboration Trials electronic databases were accessed in July 2014. Publications citing the OAST collaboration findings were also reviewed to detect potentially eligible studies. Care was taken to record only the original publication of trial results, and subsequent publications and subgroup analyses were not included.

Keywords “stroke”, “ischaemic”, “randomised”, and “Rankin” were used, accounting for differences in spelling and combination depending on the database used. The systematic review sought to include prospective, randomised, phase III studies in acute ischaemic stroke using the mRS in the primary outcome of the trial. Trials using the Oxford Handicap Scale (OHS), a very close variant of the mRS, were also included. The search was further restricted to studies published in English, from the year 2007 until July 2014. Studies of stroke prevention, haemorrhagic stroke, and those that did not involve the mRS in the primary outcome were excluded from the review.

### 2.2. Screening and Eligibility

Titles and abstracts of studies were screened in order to identify potentially eligible studies. The full texts of relevant publications were subsequently obtained and reviewed to finalise the complete list of eligible studies, excluding those that did not meet the full inclusion criteria.

### 2.3. Data Collection

Data for the primary objective of the review was collected from the full text of each publication and included the trial name, year of publication, number of randomised participants, intervention tested, and follow-up time. Additionally, the named method of analysis used in evaluation of the primary outcome measure, definition of favourable mRS outcome where applicable, and statement of the study result were also recorded.

## 3. Results

### 3.1. Study Selection

**Figure 1:**
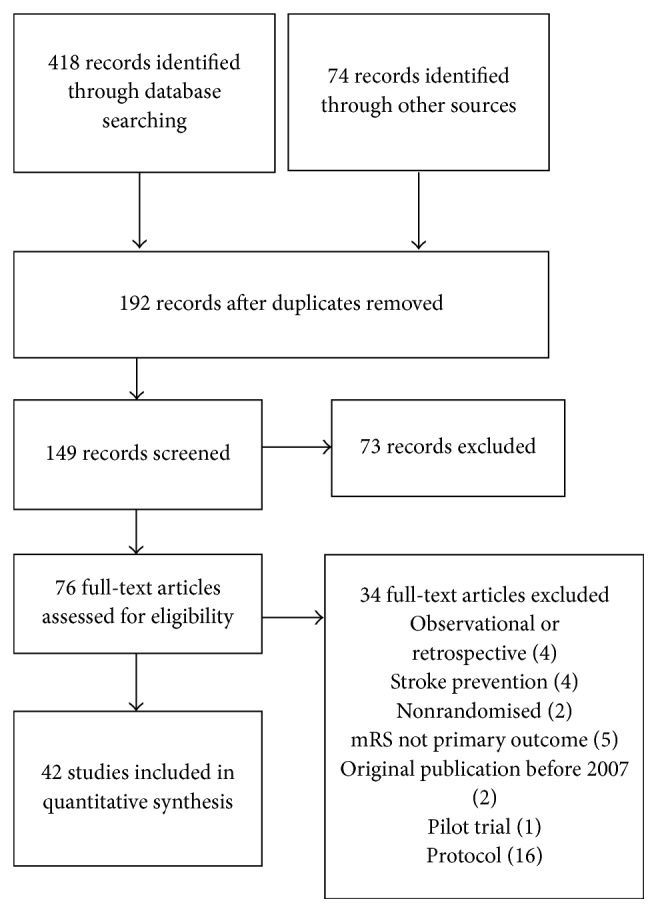
Flow of information through stages of systematic review.

A total of 192 publications were identified using the search methods after removal of duplicates. Screening of the study abstracts identified 76 potentially relevant clinical trials in ischaemic stroke using the mRS in the primary outcome (Figure 1). Eighteen studies were excluded as being nonrandomised, observational, retrospective, or pilot studies, originally published prior to 2007; trials in stroke prevention; or those not using mRS in the primary outcome.

### 3.2. Characteristics of 42 Identified Clinical Trials

A total of 42 clinical trial publications were eligible, incorporating a total of 32,432 participants, with studies ranging in size from 37 to 4,071 randomised individuals (Table 1). Nine (21.4%) trials were positive, while the vast majority of studies (31 studies, 73.8%) were unable to show benefit of the studied intervention over control. Two trials (4.8%) evaluating candesartan and statin withdrawal showed evidence of harmful intervention.

Neuroprotective or neurotrophic compounds comprised a large proportion of studied interventions in 17 (40.5%) published clinical trials. Antiplatelet or thrombolytic therapies were observed in 11 (26.2%) studies, while five (11.9%) trials sought to ameliorate physical symptoms with blood pressure management or by controlling body temperature and fever. Three (7.1%) studies investigated endovascular therapy or catheter device, while two (4.8%) sequential studies evaluated transcranial laser therapy. Three (7.1%) studies concerned the benefit of stroke rehabilitation initiatives, while one (2.4%) study examined the effect of electrical scalp acupuncture treatment.

### 3.3. Comparison of Primary Outcome Measures

Primary outcome measures differed widely across the published studies. Use of the mRS alone was observed in over half of the included studies (24 studies, 57.1%). Thirteen (31.0%) clinical trials used the mRS (or OHS) alongside other outcome measures including the Barthel Index (BI), NIH Stroke Scale (NIHSS), Quality of Life measures EQ-5D and SF-36, Glasgow Outcome Scale (GOS), Gülhane Aphasia Test (GAT), or Primary Stroke Centre (PCS) time. Five (11.9%) studies used a composite endpoint incorporating the mRS plus BI, NIHSS, or GOS scores, with three of these five studies describing a global endpoint with a threshold of result to be achieved on multiple scales.

Outcome was deemed favourable for mRS scores of 0-1 and 0–2 in equal numbers of studies, 10 (23.8%) for each. Only one (2.4%) study defined a favourable outcome to be an mRS score of 0–3. Three (7.1%) further trials defined favourable outcome scores that differed depending on baseline NIHSS score that is using a sliding dichotomy. Eighteen (35.7%) studies did not specify a desired outcome.

### 3.4. Summary of Statistical Methods Used in Individual Studies

Overall, a total of 25 (59.5%) studies used dichotomous analyses compared to eight (19.0%) studies using ordinal analyses and nine (21.4%) studies which did not fall into either category (Table 1). Tests of differences in proportions (Fisher's exact test, *χ* ^2^ test, or alternative) were employed in 14 (33.3%) studies, while binary logistic regression was used in nine (21.4%) studies, giving the advantage of producing an odds ratio, 95% confidence interval, and *p* value. Alternative modelling approaches were employed in two (4.8%) studies. Sliding dichotomy was employed in two (4.8%) studies, while a global statistic was used in two (4.8%) further trials. Statistical methods using the original ordinal scores included OLR (three studies, 7.1%) and the Cochran-Mantel-Haenszel (CMH) test (five studies, 11.9%). Four (9.5%) studies used tests based on a normal distribution (*t*-test or ordinary least squares regression) and one (2.4%) study used a Mann-Whitney *U* test.

### 3.5. Prevalence of Ordinal Methods in Secondary Analyses

Of the 11 statistically significant studies, ordinal methods were used for two (18.2%) studies. Twelve (28.6%) studies reported using ordinal methods as a secondary or sensitivity analysis. Seven (16.7%) studies used the Cochran-Mantel-Haenszel (CMH) test or van Elteren test (adjusted Mann-Whitney *U* test), while five (11.9%) studies employed OLR. Two (4.8%) studies reported the Number Needed to Treat (NNT) alongside the main trial result.

## 4. Discussion

Over half of reported studies in acute ischaemic stroke employed dichotomous analysis of an ordinal scale with wide disagreement in the threshold of favourable outcome. This result is similar to the finding by the OAST collaboration in 2007 that almost half of the 55 identified studies used a dichotomous analysis (49%), indicating that dichotomous analyses are still the prevailing choice for analysis of an ordinal scale [7]. Conversely, the OAST collaboration found around 45% of studies to employ analyses of mean or median, compared to a much smaller percentage using the same analyses in this more recent review (9.5%) [7]. Merely a fifth of studies showed significant benefit of intervention over control in this review, whereas Duncan et al. (2000) reported a systematic review of 51 studies in which a much higher percentage of studies achieved significant benefit (21 studies, 41%), although none were seen to subsequently influence clinical practice [8].

Less than a quarter of clinical trials chose to utilise analyses appropriate for an ordinal scale; however, a third of trials reported using ordinal analyses in secondary and sensitivity analyses, indicating that trial investigators were aware of these methods. Only two studies reported the NNT alongside the main trial result, despite the OAST recommendation that this measure aids clinical interpretation of the main trial result [11]. One possible explanation for this finding is how regulatory authorities, such as the FDA, authors, and journals, view ordinal analyses. The FDA has only recently accepted nondichotomous approaches for the analysis of ordinal scales. Therefore, trialists may have been reluctant to change their analysis plans while the FDA was reluctant to accept such approaches. There is anecdotal evidence to suggest that people find it hard to interpret results from ordinal analyses in terms of the clinical importance, which may also lead to hesitancy to implement these methods. Finally support for using such methods may increase as larger scale trials using such methods are published. Since the completion of this review a number of trials using an ordinal method of analysis have been published [12–15], which may encourage uptake where appropriate.

Although not shown here, we also conducted a brief scoping search of published study protocols of ongoing stroke trials. Of the published papers assessed 56% propose using an analysis preserving the ordinal scale, with six studies specifically stating that the analysis of primary outcome will be OLR, which is already numerically greater than the three published studies observed during the systematic review. Although this is a highly selective sample, it may suggest that prevalence of such methods is increasing.

Since the publication of the OAST study in 2007, there is continued interest in both developing and testing novel methods for the analysis of ordinal stroke outcomes. Use of the OLR method relies on the proportional odds assumption being met; that is, there is a common shift across cut points. Researchers should use data from previous studies to assess whether it is reasonable to assume this for the intervention being assessed. This assumption may not be met for some stroke treatments; for example, thrombolysis increases the odds of a good outcome but may, in certain circumstances, increase the odds of death. In these situations the partial proportional odds model has been advocated, where the proportional odds assumption is relaxed. This method has been shown to have some advantages over OLR when compared using data from the NINDS thrombolysis trial [16]. Assumption free alternatives have also been suggested, such as the permutation method [17]. Some have argued that another limitation of moving to an ordinal method of analysis is the interpretability of a common odds ratio [18, 19]. Therefore alterative measures of treatment effect have been proposed [20, 21], although these have had limited uptake. The NNT is a well-recognised measure of absolute treatment effect; an extension of this method for ordinal data has been suggested which may overcome this issue [11]. A limitation of these studies is that they tend to reanalyse data from one study, which makes generalisations to wider stroke trials difficult. Future research should concentrate on consolidating the extensive evidence to date on a large number of diverse trials, such as the OAST data set.

Although this review has concentrated on trials in stroke, similar work and findings have been reported in other areas, such as traumatic brain injury [22] and cancer [23]. Although based on different outcome scales the findings from the traumatic brain injury and cancer studies have generally echoed those seen in stroke. To date there has not been a review of practice in trials in these areas to assess whether there has been uptake to the methods proposed.

There are some limitations to the work presented here. Firstly, it is advised that a systematic review be conducted and data collected by two independent authors, followed by cross-checking and resolution of disagreement [24]. This review was conducted by a sole author under the supervision of a senior statistician and so it does not benefit from such validation by a second independent author. Secondly, non-English language publications were excluded from the review and as such may limit the generalisability of the findings. However, only eight non-English language papers were identified in the original list of 192 search results, and work by Morrison et al. [25] found no evidence of systematic bias in language-restricted meta-analyses; thus it is unlikely that limiting the search to English publications will have introduced bias in this review. We only included the results of published trials in this systematic review. A more comprehensive search could have also included data from completed but unpublished studies by searching trial registries such as ClinicalTrials.gov and ISRCTN [26]. There is data to suggest that published studies tend to be larger and show a greater treatment effect than those which are unpublished [27]. Therefore the studies included here may not be representative of all trials conducted during this time, and the results should be viewed with some caution.

## 5. Conclusions

The findings of this systematic review do not indicate a dramatic shift in the analysis of primary functional outcomes following acute ischaemic stroke despite the OAST recommendations; however, there appears to be awareness of the use of these methods and there may be an emerging trend towards more ordinal-appropriate analyses in ongoing and future studies.

## Figures and Tables

**Table 1:** Phase III trials in acute ischemic stroke using mRS as primary outcome published between January 2007 and July 2014.

Clinical trial (publ. year)	Intervention	Number of pts.	Primary outcome (favourable score)	Method of analysis	Result of trial
CATIS (2014)	Antihypertensive	4,071	mRS at 14 d (0–2)	*χ* ^2^ test (unadjusted), OR by logistic regression	Neutral

URICO-ICTUS (2014)	Uric acid	421	mRS at 90 d (0-1 (or 2 if premorbid score was 2))	Log-binomial regression (adjusted)	Neutral

ALIAS Part 2 (2013)	Albumin	848	mRS and NIHSS at 90 d (0-1)	GLM with log link (adjusted)	Neutral

AXIS-2 (2013)	Filgrastim (G-CSF)	328	mRS at 90 d	Ordinary least squares	Neutral

CERE-LYSE-1 (2013)^*∗*^	Cerebrolysin + alteplase	119	mRS at 90 d	Ordinal logistic regression	Neutral, trial terminated

CHIMES (Neuroaid) (2013)^*∗*^	MLC601	1,100	mRS at 3 mo	Ordinal logistic regression (adjusted)	Neutral

ECCS-AIS (2013)	Edaravone or citicoline	71	mRS and NIHSS at 3 mo	ANOVA (mean mRS score)	Positive for Edaravone

IMS III (2013)^*∗*^	Endovascular therapy	656	mRS at 3 mo (0–2)	CMH test (adjusted)	Neutral, trial stopped early

Integrated rehab (2013)	Integrated rehabilitation	69	mRS at 90 d (0-1)	Dichotomous (unavailable)	Neutral

MAC SI (2013)^*∗*^	DP-b99	446	mRS at 90 d	CMH test with modified ridit scores	Neutral (*p* = 0.105)

NBP (2013)	dl-3-n-Butylphthalide	573	mRS and BI at 90 d (0-1)	*χ* ^2^ test	Positive (*p* = 0.002)

NEST 1 & 2 pooled (2013)	Transcranial laser therapy	780	mRS at 90 d (0–2)	Logistic regression (adjusted)	Positive

SYNTHESIS Expansion (2013)	Endovascular therapy	362	mRS at 3 mo (0-1)	Fisher's exact test, OR by M-H test	Neutral

CASTA (2012)	Cerebrolysin	1,070	Global test: mRS, NIHSS, and BI at 90 d	Global directional test (Wilcoxon-Mann-Whitney test)	Neutral

Early aspirin (2012)	Aspirin + alteplase	642	mRS at 3 mo (0–2)	Dichotomous (unspecified)	Neutral, terminated early, increased risk of SICH

Ginsenoside-Rd (2012)^*∗*^	Ginsenoside-rd	390	mRS, NIHSS, BI at 90 d (0–2)	CMH test (adjusted), OR by logistic regression	Positive

Home rehabilitation (2012)	Home rehabilitation	60	mRS, BI, and EQ-5D at 2 yrs (0-1)	Dichotomous (unspecified)	Positive

ICTUS (2012)	Citicoline	2,298	global test: mRS, NIHSS, BI at 90 d	Logistic regression (adjusted)	Neutral

IST-3 (2012)	rt-PA	3,035	OHS at 6 mo (0–2)	Logistic regression (adjusted)	Neutral

Minocycline (2012)	Minocycline	50	mRS, NIHSS, BI at 90 d	*t*-test and Mann-Whitney *U* test	Positive

Scalp electrical acupuncture (2012)	Scalp electrical acupuncture	62	NIHSS, mRS, BI at postacupuncture	Fisher's exact test	Neutral

ALIAS Part 1 (2011)	Albumin	316	Composite mRS and NIHSS at 90 d (0-1)	Dichotomous (unspecified)	Neutral

Aphasia (2011)	Piracetam	49	mRS, GAT, NIHSS, and BI scores at 24 wks	*t*-test and Mann-Whitney *U* test	Neutral

CAIST (2011)	Cilostazol	458	mRS at 90 d (0–2)	Normal approximation to binomial	Comparable to aspirin (efficacy and safety)

QASC (2011)	Symptom management initiative	1,696	mRS, BI, SF-36, PSC score at 90 d (0-1)	Logistic regression with GEE	Positive

SCAST (2011)^*∗*^	Candesartan	2,029	mRS at 6 mo	Ordinal logistic regression	Negative

SENTIS (2011)	NeuroFlo device	515	Global endpoint: mRS, NIHSS, BI, and GOS at 90 d (0-1)	Logistic regression (adjusted)	Neutral

t-PA in elderly (2011)	t-PA	97	mRS at discharge (0–2)	Dichotomous (unavailable)	Neutral

COSSACS (2010)	Antihypertensive	763	mRS at 2 wks (0–2)	*χ* ^2^ test (OR by adjusted logistic regression)	Neutral, trial stopped early

EARLY (2010)^*∗*^	Aspirin + dipyridamole <24 h	548	mRS at 90 d (0-1)	CMH test (adjusted), OR by logistic regression	Neutral

ASP I & II interim (2009)	Ancrod	508	mRS at 90 d (dependent on prestroke score)	Logistic regression (adjusted)	Neutral

CHHIPS (2009)	BP manipulation	180	mRS at 2 wks (0–3)	Logistic regression	Neutral, study underpowered

DIAS-2 (2009)	90 & 125 *µ*g/kg desmoteplase	193	Composite mRS, NIHSS, and BI at 90 d	Global statistical test	Neutral

EDO (2009)	Edaravone	401	mRS at 3 mo (0-1)	Dichotomous (unavailable)	Neutral

NEST-2 (2009)	Transcranial laser therapy	660	mRS and NIHSS at 90 d (0–2)	Logistic regression (adjusted)	Neutral (*p* = 0.094)

PAIS (2009)	Paracetamol	1,400	mRS at 3 mo	Sliding dichotomy	Neutral

AbESTT-II (2008)	Abciximab	801	mRS at 3 mo	Sliding dichotomy (mRS is 0 if NIHSS is 4–7, 0-1 if 8–14, and 0–2 if 15–22)	Neutral

ECASS III (2008)	Alteplase (rt-PA)	821	mRS at 90 d (0-1)	*χ* ^2^ test (OR and RR)	Positive

Ultrasound guided TCCS (2008)	Transcranial color-coded sonography	37	mRS, BI, and death at 90 d	Mann-Whitney *U* test	Neutral mRS, overall benefit of TCCS therapy

MELT (2007)	Urokinase	114	mRS at 90 d (0–2)	Fisher's exact test	Neutral, trial stopped early

SAINT II (2007)^*∗*^	NXY-059	3,306	mRS at 90 d	CMH test (adjusted)	Neutral

Statin withdrawal (2007)	Statin withdrawal	89	mRS at 3 mo (0–2)	Logistic regression	Negative

^*∗*^Ordinal analyses. CMH: Cochran-Mantel-Haenszel, GEE: general estimating equation, GLM: generalised linear model, OR: odds ratio, and RR: relative risk.
